# Evaluating AI Scribes in Mental Health: Accuracy Across Cultures and Accents

**DOI:** 10.1192/bjo.2026.11134

**Published:** 2026-06-30

**Authors:** Adeem Tahir, Michael Seneviratne

**Affiliations:** 1 Grounded Research, Rotherham Doncaster and South Humber NHS Foundation Trust (RDaSH), Doncaster, United Kingdom; 2 University of Sheffield Medical School, Sheffield, United Kingdom; 3 Doncaster Care Group, Rotherham Doncaster and South Humber NHS Foundation Trust (RDaSH), Doncaster, United Kingdom

## Abstract

**Aims:**

The NHS Long Term Plan advocates for ambient voice technology (AVT) tools to alleviate administrative burden and improve patient access. However, psychiatric assessment relies heavily on linguistic nuance in culturally diverse presentations. Moreover, accent diversity may affect AVT speech recognition. This raises concerns about inaccurate records and the widening of health inequalities.

This study aims to evaluate the accuracy of three NHS-approved AVT tools–Company A, B, and C–in summarising psychiatric consultations across diverse cultural presentations and accents.

**Methods:**

We evaluated three AVTs using test scripts (n=3) simulating depression, dementia, and psychosis. The study included:

1. Cultural Test: Scripts adjusted for British, Nigerian, Pakistani, and Polish presentations using standardised controlled speakers.

2. Accent Test: Participants (n=9) with diverse UK and international accents reading identical scripts.

3. Output summaries were evaluated against a gold-standard checklist. Errors were classified as ‘missing/incomplete’, ‘incorrect/misleading’, or ‘hallucination’ (information not stated in the script).

**Results:**

Data are presented as mean accuracy [95% CI].

Cultural test: Company B demonstrated the highest stability: British 85% [83–87], Polish 85% [78–91], Nigerian 81% [74–88], and Pakistani 79% [72–86]. Company A displayed some variability, performing better on Nigerian 76% [69–82] and Pakistani 75% [63–86] scripts than on British 65% [43–87] and Polish 61% [34–89]. Company C consistently underperformed, ranging from British 58% [41–75] to Nigerian 56% [40–72].

Accent test: Performance was notably more stable. Company B remained stable across local (South England 84% [79–89]) and international (Spanish 80% [76–84]) speakers. Company A peaked in South England 82% [78–86] but degraded for Nigerian 69% [60–78]. Company C generally mirrored this stability (e.g. Romanian 78% [68–88]) but suffered a technical speech recognition failure for the South England accent (19% [14–23]).

Error distribution per tool: Missing/incomplete errors (>92%) dominated across all tools; 98.1% of Company A’s errors were missing/incomplete, with minimal hallucinations (1%). Company B and Company C showed higher rates of active error: Company C recorded the highest percentage of both hallucinations (2.3%) and incorrect/misleading data (5.4%), followed by Company B (1.7% and 4.7%, respectively).

**Conclusion:**

No statistically significant difference was found in overall accuracy acrosscultures or accents; however, Company B produced the most consistent summaries, while Company C produced the least. Limitations include small sample size, limited representation, and simulation design. Although seemingly robust across cultures/accents, incomplete summaries highlight the need for human oversight in clinical use. Future work must assess these tools in larger, live clinical settings to ensure safety for diverse populations.

